# Health coaching interventions for persons with chronic conditions: a systematic review and meta-analysis protocol

**DOI:** 10.1186/s13643-016-0316-3

**Published:** 2016-09-01

**Authors:** Kasey R. Boehmer, Suzette Barakat, Sangwoo Ahn, Larry J. Prokop, Patricia J. Erwin, M. Hassan Murad

**Affiliations:** 1 Mayo Clinic, 200 1st St SW, Rochester, MN 55902 USA; 2 University of Minnesota, 100 Church St. S.E., Minneapolis, MN 55455 USA

**Keywords:** Health coaching, Wellness coaching, Life coaching, Chronic disease, Chronic condition, Patient capacity

## Abstract

**Background:**

Chronic conditions are increasingly more common and negatively impact quality of life, disability, morbidity, and mortality. Health coaching has emerged as a possible intervention to help individuals with chronic conditions adopt health supportive behaviors that improve both quality of life and health outcomes.

**Methods/design:**

We planned a systematic review and meta-analysis of the contemporary health coaching literature published in the last decade to evaluate the effect of health coaching on clinically important, disease-specific, functional, and behavioral outcomes. We will include randomized controlled trials or quasi-experimental studies that compared health coaching to alternative interventions or usual care. To enable adoption of effective interventions, we aim to explore how the effect of intervention is modified by the intervention components, delivering personnel (i.e., health professionals vs trained lay or peer persons), dose, frequency, and setting. Analysis of intervention outcomes will be reported and classified using an existing theoretical framework, the Theory of Patient Capacity, to identify the areas of patients’ capacity to access and use healthcare and enact self-care where coaching may be an effective intervention.

**Discussion:**

This systematic review and meta-analysis will identify and synthesize evidence to inform the practice of health coaching by providing evidence on components and characteristics of the intervention essential for success in individuals with chronic health conditions.

**Systematic review registration:**

PROSPERO CRD42016039730

**Electronic supplementary material:**

The online version of this article (doi:10.1186/s13643-016-0316-3) contains supplementary material, which is available to authorized users.

## Background

Chronic conditions represent a growing public health problem throughout the world. In the USA, approximately half of adults have one or more chronic condition [1], while 26 % have multiple chronic conditions. As the population ages, multiple chronic conditions will affect 3 in 4 Americans 65 and older [2, 3]. Chronic conditions decrease quality of life, increase disability, increase morbidity and mortality, and increase healthcare costs.

Health coaching has emerged as a widely adopted intervention to help individuals with chronic conditions adopt health supportive behaviors that improve both quality of life and health outcomes. Commonly referred to as “life coaching,” “health coaching,” or “wellness coaching,” the lack of definitional clarity has made it difficult to study and compare coaching interventions. The coaching process is viewed as “a systematic process and is typically directed at fostering the ongoing self-directed learning and personal growth of the client” [4]. A comprehensive conceptual definition of health coaching was provided by Wolever et al. who defines coaching as “a patient-centered approach wherein patients at least partially determine their goals, use self-discovery or active learning processes together with content education to work toward their goals, and self-monitor behaviors to increase accountability, all within the context of an interpersonal relationship with a coach” [5]. There are several features common to nearly all forms of coaching. Commonalities include the core assumption that people have an innate capacity to grow and develop; a focus on constructing solutions; and a focus on goal attainment processes [4].

There is a need for evidence synthesis to evaluate the effectiveness of health coaching, particularly to examine the components that are necessary for its effectiveness and settings in which it is most helpful. Such synthesis can provide evidence of the effectiveness of health coaching that cuts across types and models of coaching. For example, prior to the publication of Wolever et al.’s conceptual definition, two health coaching systematic reviews made exclusions based on specific labels or narrow definitions of coaching traditions. Kivela et al. explored “life coaching,” thereby excluding interventions labeled “health coaching,” while Ammentorp et al. explored strategies labeled “health coaching” at the expense of excluding interventions labeled “life coaching” or “wellness coaching” [4, 6]. Therefore, we plan to conduct a systematic review and meta-analysis that further builds on previous systematic reviews by including coaching interventions based on a broad conceptual definition of health coaching [5].

Unlike previous syntheses, we also plan to include interventions delivered by lay and peer-coaches. The review by Kivela et al. purposefully excludes coaching interventions not delivered by a health professional [6]. However, Wolever et al. found that approximately 6 % of coaching interventions used lay coaches while another 13 % did not provide sufficient information to determine the coaches’ background [5]. Therefore, including coaching provided by the growing segment of lay and peer-coaches will provide knowledge about their application and comparison to health professional-delivered coaching [7–12].

Additionally, the planned review seeks to clarify the characteristics of coaching delivery that make it most effective by identifying the ideal coaching duration, frequency, delivery format, and coach qualifications for individuals and subsets of individuals with chronic conditions. This information will help communities, clinics, health coaches, and healthcare systems to create successful, effective health coaching interventions tailored to the needs of those with chronic conditions.

This review will pay particular attention to the impact of health coaching on outcomes that contribute to patient capacity to access and use healthcare and enact self-care. Patient capacity has important implications for health outcomes and the experience of care. Individuals experience higher treatment burdens when they have limited capacity to manage emotional problems with family and friends, role and activity limitations, financial challenges, and healthcare delivery inefficiencies [13]. Furthermore, patients draw on capacity to make adaptations over time to overcome treatment burden associated with chronic conditions [14]. Studies that have examined capacity have shown that patients most disrupted by treatment had serious limitations to their physical, emotional, and financial capacities [15] while care plans that attended to patient capacity were more effective in reducing 30-day hospital readmissions [16].

Given health coaching’s potential to improve health outcomes by supporting and growing patients’ capacity to cope with the demands of chronic illness, this review will highlight outcomes related to health coaching’s effect on patient capacity using the descriptive Theory of Patient Capacity. The Theory of Patient Capacity maintains that factors that shape patient capacity include the successful reframing of their *Biography*, the ability to mobilize new or existing *Resources*, the support of their *Environment*, their experiential accomplishment of patient and life *Work*, and their *Social* functioning (BREWS) [17]. Coaching as an intervention naturally explores and cultivates human capacity in each of these areas. Since increasing patient capacity is now appreciated as a strategy to improve patient health outcomes, we would like to elucidate the relationship between health coaching and increasing patient capacity. This relationship may reveal an opportunity to refine a health coaching approach to focus on Capacity Coaching in patients with chronic conditions and large treatment burdens.

## Aim

We aim to explore clinically important outcomes of health coaching interventions for persons with chronic conditions applying a broad conceptual definition of health coaching and including peer and lay-health coaching interventions. Comparators will include usual care, therapy interventions, health education, and support groups.

Important outcomes for patients’ health will include disease-specific and physiologic outcomes, behavioral and psychological outcomes, and measures of patient functioning (ADLs). All outcomes that play a role in patients’ capacity, as described by Theory of Patient Capacity, will be categorized using the BREWS framework.

This review further aims to identify the characteristics of coaching delivery that make it most effective for individuals with chronic conditions by identifying the ideal coaching duration, frequency, delivery format and coach qualifications, involvement of the primary care provider, for individuals or subsets of individuals with chronic conditions.

## Methods

### Study design

We will conduct a systematic review and meta-analysis that adheres to the reporting guidelines of the Preferred Reporting Items for Systematic Reviews and Meta-Analyses (PRISMA) statement [18]. We have developed this protocol in accordance with the PRISMA-P statement, which is included as an additional file (Additional file 1) [19].

### Study eligibility

#### Types of studies

We will include studies published in English, which use a randomized controlled trial or quasi-experimental design to compare health coaching to standard care or other alternative interventions.

#### Types of participants

We will include studies that seek to apply coaching as an intervention for adults aged 18 and older with one or more chronic conditions. We will use the Agency for Healthcare Quality and Research’s (AHRQ) definition of a chronic condition as one “lasting 12 months or more and limiting self-care, independent living, or requires ongoing medical intervention” [20]. Coaching interventions that are delivered in a healthcare setting but participants do not have a chronic condition will be excluded. For example, coaching services could be provided to patients receiving primary care at a health clinic, who are otherwise healthy, for general health maintenance, stress management, etc.

#### Types of interventions

We will include studies that employ coaching interventions for individuals with chronic conditions. These interventions may be delivered in-person, by telephone, by internet, or a combination of multiple delivery methods. The interventions may be delivered individually to patients, as part of a group, or a combination. We will seek as much information about the intervention from the papers retrieved to determine if it meets definition of coaching presented by Wolever et al. for inclusion [5]. Specifically, in order to meet inclusion under this definition, interventions must (1) be patient-centered in that patients must at least partially determine their goals; (2) use self-discovery or active learning processes together with content education to work toward patient goals; (3) have a component of behavioral self-monitoring to increase accountability; and (4) pursue these activities in the context of an interpersonal relationship with a coach [5]. Finally, while Wolever et al. specifies that health professionals use these strategies, we are interested in the delivery of coaching interventions by health professionals as well as those that are outside traditional healthcare roles, which may include community health workers or peer-coaches, which, as indicated above have become increasingly common in the recent literature.

#### Types of comparators

We will include studies that compare coaching to standard clinical care or alternative intervention(s). Alternative interventions to which coaching may be compared may include but are not limited to patient education interventions, counseling, support groups, or chronic disease self-management courses. These interventions may have individual components included in the Wolever definition of coaching but do not satisfy all four criteria. For example, patient education interventions may include content education in the context of an interpersonal relationship but not include any self-discovery or goal-setting.

#### Types of outcome measures

We will extract three types of outcomes: (1) disease-specific (preferably patient important outcomes although surrogate outcomes are more likely be pursued in health coaching trials), (2) outcomes that relate to patient functioning and quality of life such as the ability to perform activities of daily living (ADLs), and (3) behavioral and psychosocial outcomes.

Some of these outcomes will be disease-specific and some will be common across diseases, particularly those that relate to function and quality of life. Because we are interested in coaching as a mechanism to improve patient capacity, where feasible we will categorize outcomes using the Theory of Patient Capacity as a guide into outcomes that shape related to patient reframing of their *Biography* (i.e., role function, quality of life), the ability to mobilize new or existing *Resources* (i.e., physical function, activity limitation), the support of their *Environment* (i.e., healthcare team support), their experiential accomplishment of patient and life *Work* (i.e., self-efficacy, patient activation), and their *Social* functioning (i.e., social support) [17].

#### Search strategy

Two expert reference librarians (LP, PJE) will create and conduct the initial search of relevant search databases including Ovid MEDLINE, Ovid EMBASE, CINAHL, Ovid PsycINFO, Ovid Cochrane CENTRAL, and Scopus. This search will include studies from February 2006 to February 2016 that include key terms such as coach, wellness, health promotion, health behavior, lifestyle, peer, recover and chronic disease, as well as disease-specific search terms that we have used in previous reviews [17] for a more sensitive search of all chronic conditions. We will review the references of included studies to ensure that no additional studies should be included. A full search strategy is included as an appendix to this protocol (Additional file 2).

#### Selection of studies

Duplicates are removed by the librarians (LP, PJE), using EndNote’s duplicate identification strategy and then manually. When reviewing and retrieving citations, any missing citation data is located, or added manually. After removing duplicates, we will import retained studies into systematic review software (DistillerSR, Ottawa, ON, Canada). We will screen studies in two phases: abstract screening and full-text screening. In each phase, three authors (KB, SB, and SA) will undergo training to ensure clarity about study purpose, inclusion, and exclusion criteria and then screen a subset of abstracts in triplicate to ensure good inter-rater agreement. After this step, in both phases, each abstract or full text will be screened individually and in duplicate by two of the trained reviewers. In the abstract screening phase, both reviewers must be in agreement in order to exclude an article; conflicts will be included. During full-text screening, conflicts will be resolved by consensus. If consensus cannot be achieved between the two reviewers, the third reviewer will arbitrate.

#### Data extraction

Data will be extracted using the same systematic review software used for abstract and full-text screening (DistillerSR, Ottawa, ON, Canada). We have developed and pilot tested the extraction form to ensure effective and efficient data extraction. Data extraction will be performed in duplicate. Paired reviewers will review data extraction conflicts by reviewing studies to correct errors. Any extraction conflicts not easily resolved by re-review of the full text will be resolved by consensus. We will extract lead author names, date, country in which the study was conducted, chronic condition(s) targeted, study aim, study design, sample subjects characteristics including total number, age, sex, loss to follow-up, ethnicity/race, sample income information, intervention and control characteristics including the person delivering the intervention, number of sessions prescribed, duration of session prescribed (minutes), mean number of sessions (actual), mean duration of sessions (actual), length of intervention (actual, in weeks), coaching/theoretical model(s), setting, description of the coaching (goal-setting, relationship with coach, etc),whether it is a multi-component intervention, whether a clinician was involved in the intervention, and control group characteristics. We will note all outcome measures related to the patient’s health or their capacity to access and use healthcare and enact self-care, when each outcome measure was collected during the study, measures used to collect outcomes, and main findings. These include, but are not limited to, outcomes such as quality of life, global health status, activity limitation, pain, depression, anxiety, HbA1c, body mass index (BMI), self-efficacy, patient activation, physical activity, social support, and mental health.

#### Risk of bias assessment

We will assess risk of bias using the Cochrane Collaboration’s tool for assessing risk of bias, which prompts assessment in the following areas: randomization, quality of randomization (any important imbalances at baseline), allocation concealment, level(s) of blinding, incomplete outcome data (due to attrition or otherwise), selective reporting, and other sources of bias, considering other factors such as conducting intention to treat analysis and the role of funding in the study [21]. We will include a study-level table that describes the risk of bias in these categories for each study and describe the risk of bias in the overall body of evidence. No studies will be excluded based on the risk of bias assessment.

#### Statistical analysis

As a general framework, we plan to conduct meta-analysis using a random-effects model. We chose this model a priori to incorporate within-study and between-study heterogeneity since we anticipate difference across studies population and interventions. If the number of studies in a particular analysis is large (15–20), we will use the DerSimonian Laird method [22]; if the number is smaller, we will use the Knapp–Hartung small-sample estimator approach method [23]. We will evaluate heterogeneity using the *I*^2^ statistic, *τ*^2^, and the Cochrane Q statistic. Substantial heterogeneity will be defined as *I*^2^ larger than 50 % or Q test with a *p* value <0.10. It is likely that certain outcomes will be measured using different scales or various definitions and would require standardization. In this instance, we would estimate the standardized effect size and present the effect of wellness coaching in standard deviation units. If we have more than 20 studies per analysis, we will construct funnel plots and test their symmetry using one of the several available techniques that test for small study effect (e.g., Egger’s regression). Unfortunately, all these methods have serious limitations [24].

#### Exploring heterogeneity

Since wellness coaching is a complex intervention by nature, we will follow suggestions on synthesizing evidence from complex interventions [25]. Briefly, such framework emphasizes that evidence users are more interested in knowing when the intervention works (delivered by whom and how and how often, and in which setting and patient subgroup). Such knowledge is more important than the simple question of “does it work?” Therefore, we plan to conduct univariate and multivariate meta-regression to explore the effect of such characteristics (the independent variables) on the effect size (the dependent variable). This exploration of heterogeneity may help in identifying factors, components, and conditions in which health coaching is more effective and, therefore, may facilitate implementation of such effective components of the intervention. Other key characteristics of the population and setting (e.g., diabetes) will also be explored as possible covariates. Analyses will be performed in Stata, version 13 (StataCorp). Using the (Metareg) command, the effect size will be the dependent variable and the complex intervention characteristics will be the independent variables. The statistics obtained from the random permutations can be used to adjust for such multiple testing by comparing the observed t-statistic for every covariate with the largest t-statistic for any covariate in each random permutation. We will produce a bubble graph showing the fitted regression line [26]. We will perform a quantitative analysis when possible (i.e., when we expect that studies have similar population, intervention, comparator and outcomes; to the extent that a similar effect size is anticipated if studies were pooled). We will pursue a narrative analysis when statistical analysis is not an option.

#### Quality of evidence

We will evaluate quality of evidence (certainty of evidence) using the GRADE approach [27]. Quality of evidence from randomized trials starts at high and can be rated down for methodological limitation, imprecision, indirectness, inconsistency, or publication bias. Quasi-experimental studies may start at low if they were clearly nonrandomized [28].

## Discussion

Chronic conditions are the leading causes of morbidity and mortality in the USA and across the world. A new public health approach requires efforts in the realms of public health policy, community based programs, and clinical preventive services [29]. Based on a growing body of evidence that health coaching can improve health outcomes for individuals with chronic conditions, health coaching as an individual or group intervention may support public health efforts in the realms of community-based programs and clinical preventive services [4, 6].

While previous reviews have captured the effects of health coaching on various health outcomes, we hope to substantiate this work with a more inclusive approach that includes all coaching interventions and the growing body of work performed by peer and lay coaches. While the strength of this review is its considerable depth of information and broad inclusion criteria, its potential limitations must be considered. We expect that there may be limitations to the evidence summary and synthesis, related to poor indexing of the coaching literature and lack of reporting of the characteristics of interventions, to the extent that meta-regression may not be feasible. Caution in the interpretation of meta-regression is needed since misleading associations can be observed due to ecological bias and confounding. Lastly, operationalization of the “BREWS” framework will be iterative during the conduct of this review since this approach is novel and therefore analysis methods would be exploratory.

**Fig. 1:**
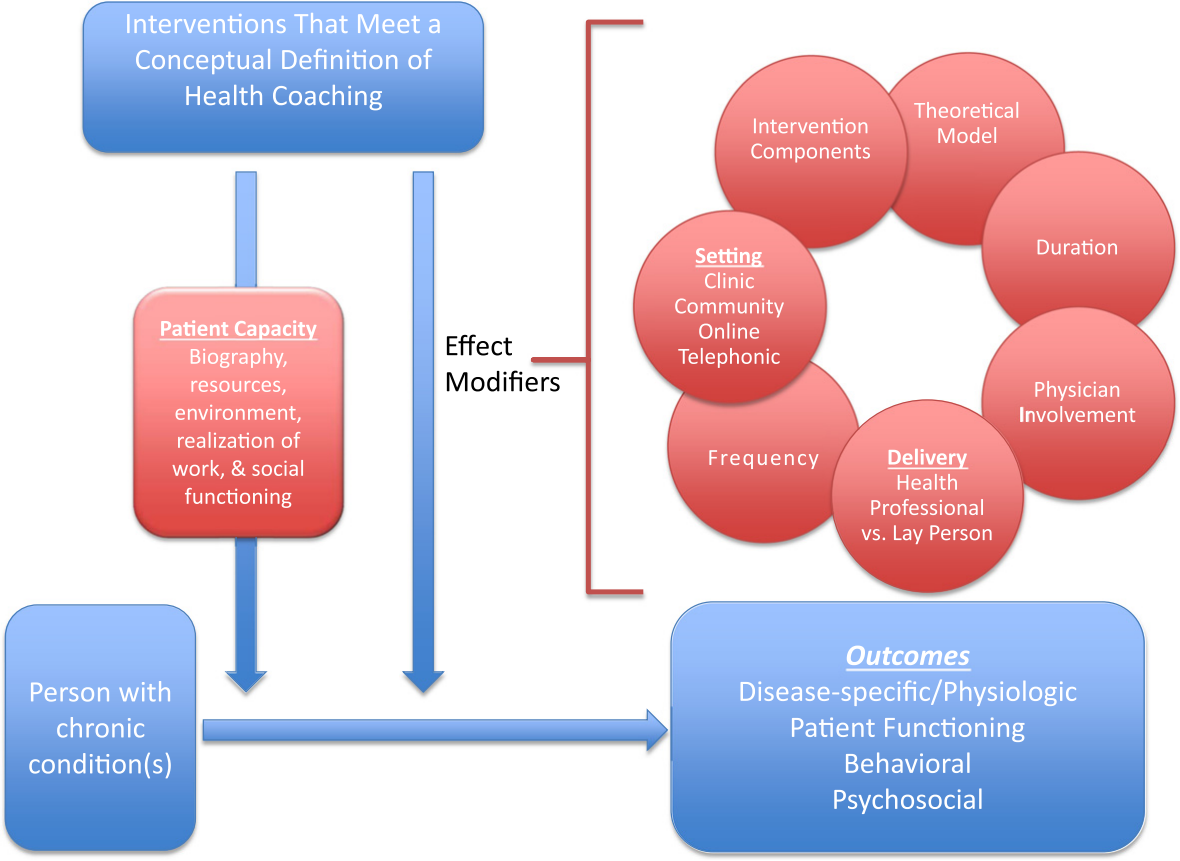
Health coaching analytic framework

Finally, our novel sub-focus on the relationship between health coaching and patient capacity will elucidate any basis to the hypothesis that we can enhance health in chronic conditions by focusing on developing a sub-category of coaching, deemed “Capacity Coaching.” This is in line with the extensive body of research growing to support the practice of Minimally Disruptive Medicine [13–15, 30–32], of which Capacity Coaching would be a component. Finally, to make it easier to design and implement effective health coaching interventions in support of the 66 % of Americans with chronic conditions, this review seeks to identify key characteristics of successful coaching interventions. In addition to informing coaching practice broadly, Capacity Coaching will incorporate the knowledge gained from this review with its theoretical underpinnings, to better inform its model with ideal format, timing, training, and delivery personnel (Fig. 1).

Overall this review will support clinicians, nurses, clinics, healthcare systems, health plans, community health organizations and public health departments as they consider scalable health coaching interventions for individuals with chronic conditions. As we gain insights about the elements of effective health coaching interventions that optimally increase patient capacity to self manage chronic conditions, we strengthen the medicine-public health partnership needed to manage and reverse the chronic disease trend.

## Additional files

Additional file 1: PRISMA-P checklist acknowledges we have met key reporting items for Systematic review and Meta-Analysis Protocols. (DOC 84 kb) Additional file 2: Literature search strategies. Includes full search strategies used in the systematic review. (DOCX 34 kb)

## Abbreviations

ADLs: Activities of daily living
AHRQ: Agency for Healthcare Quality and Research
BMI: Body mass index
BREWS: Biography, resources, environment, work, and social functioning
