# Supplementary material for: Health coaching interventions for persons with chronic conditions: a systematic review and meta-analysis protocol
Source: Syst Rev. 2016 Sep 1;5(1):146. doi: 10.1186/s13643-016-0316-3 (PMC5009492; doi:10.1186/s13643-016-0316-3)
Supplement: Additional file 1: — PRISMA-P checklist acknowledges we have met key reporting items for Systematic review and Meta-Analysis Protocols. (DOC 84 kb) [file 13643_2016_316_MOESM1_ESM.doc]

**PRISMA-P (Preferred Reporting Items for Systematic review and Meta-Analysis Protocols) 2015 checklist: recommended items to address in a systematic review protocol***

| Section and topic | Item No | Checklist item |
| --- | --- | --- |
| ADMINISTRATIVE INFORMATION | | |
| Title: |  | Health coaching interventions for persons with chronic conditions: A systematic review and meta-analysis protocol |
| Identification | 1a | Identify the report as a protocol of a systematic review |
| Update | 1b | If the protocol is for an update of a previous systematic review, identify as such |
| Registration | 2 | PROSPERO registration number: CRD42016039730 |
| Authors: |  | Kasey Boehmer, [boehmer.kasey@mayo.edu](mailto:boehmer.kasey@mayo.edu), Mayo Clinic, 200 1st St SW, Rochester, MN 55902  Suzette Lee Barakat MD, barakat.suzette@mayo.edu, Mayo Clinic, 200 1st St SW, Rochester, MN 55902  Sangwoo Ahn, [ahnxx230@umn.edu](mailto:ahnxx230@umn.edu), University of Minnesota,100 Church St. S.E. Minneapolis MN 55455  Larry Prokop, MLS. prokop.larry@mayo.edu, Mayo Clinic, 200 1st St SW, Rochester, MN 55902  Patricia J Erwin, MLS. erwin.patricia@mayo.edu, Mayo Clinic, 200 1st St SW, Rochester, MN 55902  M. Hassan Murad, [Hassan.murad@mayo.edu](mailto:Hassan.murad@mayo.edu), Mayo Clinic, 200 1st St SW, Rochester, MN 5590 |
| Contact | 3a | Provide name, institutional affiliation, e-mail address of all protocol authors; provide physical mailing address of corresponding author.  Provided above. |
| Contributions | 3b | Describe contributions of protocol authors and identify the guarantor of the review: KRB and SLB conceptualized the scope of the review and study design with mentorship from MHM. LP and PJE developed the search strategies. KRB, SLB, and MHM serve as guarantors of this review. |
| Amendments | 4 | NONE  If the protocol represents an amendment of a previously completed or published protocol, identify as such and list changes; otherwise, state plan for documenting important protocol amendments |
| Support: |  | Sources/Sponsor: Financial Support from Mayo Clinic to cover cost of submitting for publication and for obtaining full text articles through interlibrary loan.  Role of Sponsor/funder: none |
| Sources | 5a | Indicate sources of financial or other support for the review  None |
| Sponsor | 5b | Provide name for the review funder and/or sponsor  None |
| Role of sponsor or funder | 5c | Describe roles of funder(s), sponsor(s), and/or institution(s), if any, in developing the protocol  None |
| INTRODUCTION | | |
| Rationale | 6 | Describe the rationale for the review in the context of what is already known  Pages 3-5 |
| Objectives | 7 | Provide an explicit statement of the question(s) the review will address with reference to participants, interventions, comparators, and outcomes (PICO)  Page 6 |
| METHODS | | |
| Eligibility criteria | 8 | Specify the study characteristics (such as PICO, study design, setting, time frame) and report characteristics (such as years considered, language, publication status) to be used as criteria for eligibility for the review  Pages 7-9 |
| Information sources | 9 | Describe all intended information sources (such as electronic databases, contact with study authors, trial registers or other grey literature sources) with planned dates of coverage  Page 9 |
| Search strategy | 10 | Present draft of search strategy to be used for at least one electronic database, including planned limits, such that it could be repeated  Supplementary Materials |
| Study records: |  |  |
| Data management | 11a | Describe the mechanism(s) that will be used to manage records and data throughout the review  Pages 9-10 |
| Selection process | 11b | State the process that will be used for selecting studies (such as two independent reviewers) through each phase of the review (that is, screening, eligibility and inclusion in meta-analysis)  Pages 9-10 |
| Data collection process | 11c | Describe planned method of extracting data from reports (such as piloting forms, done independently, in duplicate), any processes for obtaining and confirming data from investigators  Page 10 |
| Data items | 12 | List and define all variables for which data will be sought (such as PICO items, funding sources), any pre-planned data assumptions and simplifications  Page 10 |
| Outcomes and prioritization | 13 | List and define all outcomes for which data will be sought, including prioritization of main and additional outcomes, with rationale  Page 10 |
| Risk of bias in individual studies | 14 | Describe anticipated methods for assessing risk of bias of individual studies, including whether this will be done at the outcome or study level, or both; state how this information will be used in data synthesis  Page 11 |
| Data synthesis | 15a | Describe criteria under which study data will be quantitatively synthesised  Pages 12-13. |
|  | 15b | If data are appropriate for quantitative synthesis, describe planned summary measures, methods of handling data and methods of combining data from studies, including any planned exploration of consistency (such as I^2^, Kendall’s τ)  Pages 13 |
|  | 15c | Describe any proposed additional analyses (such as sensitivity or subgroup analyses, meta-regression)  Pages 13. |
|  | 15d | If quantitative synthesis is not appropriate, describe the type of summary planned  Page 12-13 |
| Meta-bias(es) | 16 | Specify any planned assessment of meta-bias(es) (such as publication bias across studies, selective reporting within studies) |
|  |  | Page 13 |
| Confidence in cumulative evidence | 17 | Describe how the strength of the body of evidence will be assessed (such as GRADE)  Page 14 |

*** It is strongly recommended that this checklist be read in conjunction with the PRISMA-P Explanation and Elaboration (cite when available) for important clarification on the items. Amendments to a review protocol should be tracked and dated. The copyright for PRISMA-P (including checklist) is held by the PRISMA-P Group and is distributed under a Creative Commons Attribution Licence 4.0.**

*From: Shamseer L, Moher D, Clarke M, Ghersi D, Liberati A, Petticrew M, Shekelle P, Stewart L, PRISMA-P Group. Preferred reporting items for systematic review and meta-analysis protocols (PRISMA-P) 2015: elaboration and explanation. BMJ. 2015 Jan 2;349(jan02 1):g7647.*
