# Supplementary material for: Health coaching interventions for persons with chronic conditions: a systematic review and meta-analysis protocol
Source: Syst Rev. 2016 Sep 1;5(1):146. doi: 10.1186/s13643-016-0316-3 (PMC5009492; doi:10.1186/s13643-016-0316-3)
Supplement: Additional file 2: — Literature search strategies. Includes full search strategies used in the systematic review. (DOCX 34 kb) [file 13643_2016_316_MOESM2_ESM.docx]

| **Ovid MEDLINE(R) In-Process & Other Non-Indexed Citations and Ovid MEDLINE(R)**1946 to Present | | | |
| --- | --- | --- | --- |
| **#** | **Searches** | **Results** | **Search Type** |
| 1 | chronic disease/ or chronic*.mp. [mp=title, abstract, original title, name of substance word, subject heading word, keyword heading word, protocol supplementary concept word, rare disease supplementary concept word, unique identifier] | 1079570 | Advanced |
| 2 | 1 and coach*.mp. [mp=title, abstract, original title, name of substance word, subject heading word, keyword heading word, protocol supplementary concept word, rare disease supplementary concept word, unique identifier] | 485 | Advanced |
| 3 | *diabetes mellitus/ or *diabetes mellitus, type 1/ or *diabetes mellitus, type 2/ or diabet*.mp. [mp=title, abstract, original title, name of substance word, subject heading word, keyword heading word, protocol supplementary concept word, rare disease supplementary concept word, unique identifier] | 518406 | Advanced |
| 4 | *hypertension/ or "blood pressure".mp. or hypertens*.mp. [mp=title, abstract, original title, name of substance word, subject heading word, keyword heading word, protocol supplementary concept word, rare disease supplementary concept word, unique identifier] | 661383 | Advanced |
| 5 | (copd or "chronic obstructive").mp. or exp *pulmonary disease, chronic obstructive/ or asthma*.mp. or exp *asthma/ [mp=title, abstract, original title, name of substance word, subject heading word, keyword heading word, protocol supplementary concept word, rare disease supplementary concept word, unique identifier] | 200315 | Advanced |
| 6 | *arthritis/ or exp *arthritis, rheumatoid/ or *osteoarthritis/ or arthrit*.mp. or osteoarthr*.mp. [mp=title, abstract, original title, name of substance word, subject heading word, keyword heading word, protocol supplementary concept word, rare disease supplementary concept word, unique identifier] | 243470 | Advanced |
| 7 | exp *epilepsy/ or exp *obesity/ or exp *liver diseases/ or exp *kidney diseases/ or exp *heart diseases/ or exp *hiv infections/ or exp *neoplasms/ or exp *stroke/ | 4167036 | Advanced |
| 8 | or/3-7 | 5403753 | Advanced |
| 9 | 8 and coach*.mp. [mp=title, abstract, original title, name of substance word, subject heading word, keyword heading word, protocol supplementary concept word, rare disease supplementary concept word, unique identifier] | 1010 | Advanced |
| 10 | 2 or 9 | 1312 | Advanced |
| 11 | (epilep* or obese or obesity or liver or hepat* or kidney or renal or nephr* or heart or cardiac or coronary or myocard* or hiv or cancer or stroke*).mp. [mp=title, abstract, original title, name of substance word, subject heading word, keyword heading word, protocol supplementary concept word, rare disease supplementary concept word, unique identifier] | 5118328 | Advanced |
| 12 | 11 and coach*.mp. [mp=title, abstract, original title, name of substance word, subject heading word, keyword heading word, protocol supplementary concept word, rare disease supplementary concept word, unique identifier] | 1537 | Advanced |
| 13 | 12 and (health promotion/ or behavior*.mp. or behaviour*.mp. or change*.mp. or lifestyle.mp. or "life style".mp.) [mp=title, abstract, original title, name of substance word, subject heading word, keyword heading word, protocol supplementary concept word, rare disease supplementary concept word, unique identifier] | 615 | Advanced |
| 14 | ((life or lifestyle or health or behavior* or wellness or peer or recover*) adj3 coach*).mp. [mp=title, abstract, original title, name of substance word, subject heading word, keyword heading word, protocol supplementary concept word, rare disease supplementary concept word, unique identifier] | 789 | Advanced |
| 15 | (8 or 11 or chronic*.mp.) and 14 [mp=title, abstract, original title, name of substance word, subject heading word, keyword heading word, protocol supplementary concept word, rare disease supplementary concept word, unique identifier] | 367 | Advanced |
| 16 | 10 or 13 or 15 | 1594 | Advanced |
| 17 | limit 16 to yr="2005 - 2016" | 1240 | Advanced |
| 18 | 17 and (study or studies or cohort* or intervention* or prospective* or retrospective* or "cross section*").mp. [mp=title, abstract, original title, name of substance word, subject heading word, keyword heading word, protocol supplementary concept word, rare disease supplementary concept word, unique identifier] | 1004 | Advanced |
| 19 | 17 and random*.mp. [mp=title, abstract, original title, name of substance word, subject heading word, keyword heading word, protocol supplementary concept word, rare disease supplementary concept word, unique identifier] | 452 | Advanced |
| 20 | limit 17 to (clinical study or clinical trial, all or clinical trial, phase i or clinical trial, phase ii or clinical trial, phase iii or clinical trial, phase iv or clinical trial or comparative study or controlled clinical trial or evaluation studies or meta analysis or multicenter study or observational study or pragmatic clinical trial or randomized controlled trial or systematic reviews or validation studies) | 498 | Advanced |
| 21 | 18 or 19 or 20 | 1021 | Advanced |
| 22 | remove duplicates from 21 | 1013 |  |

CENTRAL – same strategy = 416

| **Embase**1988 to 2016 Week 05 | | | |
| --- | --- | --- | --- |
| **#** | **Searches** | **Results** | **Search Type** |
| 1 | chronic disease/ or chronic*.mp. [mp=title, abstract, heading word, drug trade name, original title, device manufacturer, drug manufacturer, device trade name, keyword] | 1218229 | Advanced |
| 2 | 1 and coach*.mp. [mp=title, abstract, heading word, drug trade name, original title, device manufacturer, drug manufacturer, device trade name, keyword] | 722 | Advanced |
| 3 | *diabetes mellitus/ or *diabetes mellitus, type 1/ or *diabetes mellitus, type 2/ or diabet*.mp. [mp=title, abstract, heading word, drug trade name, original title, device manufacturer, drug manufacturer, device trade name, keyword] | 726070 | Advanced |
| 4 | *hypertension/ or "blood pressure".mp. or hypertens*.mp. [mp=title, abstract, heading word, drug trade name, original title, device manufacturer, drug manufacturer, device trade name, keyword] | 802256 | Advanced |
| 5 | (copd or "chronic obstructive").mp. or exp *pulmonary disease, chronic obstructive/ or asthma*.mp. or exp *asthma/ [mp=title, abstract, heading word, drug trade name, original title, device manufacturer, drug manufacturer, device trade name, keyword] | 282798 | Advanced |
| 6 | *arthritis/ or exp *arthritis, rheumatoid/ or *osteoarthritis/ or arthrit*.mp. or osteoarthr*.mp. [mp=title, abstract, heading word, drug trade name, original title, device manufacturer, drug manufacturer, device trade name, keyword] | 287109 | Advanced |
| 7 | exp *epilepsy/ or exp *obesity/ or exp *liver diseases/ or exp *kidney diseases/ or exp *heart diseases/ or exp *hiv infections/ or exp *neoplasms/ or exp *stroke/ | 3661981 | Advanced |
| 8 | or/3-7 | 5177764 | Advanced |
| 9 | 8 and coach*.mp. [mp=title, abstract, heading word, drug trade name, original title, device manufacturer, drug manufacturer, device trade name, keyword] | 1686 | Advanced |
| 10 | 2 or 9 | 2058 | Advanced |
| 11 | (epilep* or obese or obesity or liver or hepat* or kidney or renal or nephr* or heart or cardiac or coronary or myocard* or hiv or cancer or stroke*).mp. [mp=title, abstract, heading word, drug trade name, original title, device manufacturer, drug manufacturer, device trade name, keyword] | 6095514 | Advanced |
| 12 | 11 and coach*.mp. [mp=title, abstract, heading word, drug trade name, original title, device manufacturer, drug manufacturer, device trade name, keyword] | 2230 | Advanced |
| 13 | 12 and (health promotion/ or behavior*.mp. or behaviour*.mp. or change*.mp. or lifestyle.mp. or "life style".mp.) [mp=title, abstract, heading word, drug trade name, original title, device manufacturer, drug manufacturer, device trade name, keyword] | 1010 | Advanced |
| 14 | ((life or lifestyle or health or behavior* or wellness or peer or recover*) adj3 coach*).mp. [mp=title, abstract, heading word, drug trade name, original title, device manufacturer, drug manufacturer, device trade name, keyword] | 1102 | Advanced |
| 15 | (8 or 11 or chronic*.mp.) and 14 [mp=title, abstract, heading word, drug trade name, original title, device manufacturer, drug manufacturer, device trade name, keyword] | 591 | Advanced |
| 16 | 10 or 13 or 15 | 2471 | Advanced |
| 17 | limit 16 to yr="2005 - 2016" | 2107 | Advanced |
| 18 | 17 and (study or studies or cohort* or intervention* or prospective* or retrospective* or "cross section*").mp. [mp=title, abstract, heading word, drug trade name, original title, device manufacturer, drug manufacturer, device trade name, keyword] | 1700 | Advanced |
| 19 | 17 and random*.mp. [mp=title, abstract, heading word, drug trade name, original title, device manufacturer, drug manufacturer, device trade name, keyword] | 667 | Advanced |
| 20 | clinical study/ or exp case control study/ or exp case study/ or exp clinical trial/ or exp intervention study/ or exp major clinical study/ or exp prospective study/ or exp retrospective study/ | 3193202 | Advanced |
| 21 | 17 and 20 | 706 | Advanced |
| 22 | 18 or 19 or 21 | 1735 | Advanced |
| 23 | limit 22 to (adult <18 to 64 years> or aged <65+ years>) | 687 |  |

| **PsycINFO**1987 to February Week 1 2016 | | | |
| --- | --- | --- | --- |
| **#** | **Searches** | **Results** | **Search Type** |
| 1 | chronic disease/ or chronic*.mp. [mp=title, abstract, heading word, table of contents, key concepts, original title, tests & measures] | 111758 | Advanced |
| 2 | 1 and coach*.mp. [mp=title, abstract, heading word, table of contents, key concepts, original title, tests & measures] | 198 | Advanced |
| 3 | *diabetes mellitus/ or *diabetes mellitus, type 1/ or *diabetes mellitus, type 2/ or diabet*.mp. [mp=title, abstract, heading word, table of contents, key concepts, original title, tests & measures] | 22556 | Advanced |
| 4 | *hypertension/ or "blood pressure".mp. or hypertens*.mp. [mp=title, abstract, heading word, table of contents, key concepts, original title, tests & measures] | 23002 | Advanced |
| 5 | (copd or "chronic obstructive").mp. or exp *pulmonary disease, chronic obstructive/ or asthma*.mp. or exp *asthma/ [mp=title, abstract, heading word, table of contents, key concepts, original title, tests & measures] | 7120 | Advanced |
| 6 | *arthritis/ or exp *arthritis, rheumatoid/ or *osteoarthritis/ or arthrit*.mp. or osteoarthr*.mp. [mp=title, abstract, heading word, table of contents, key concepts, original title, tests & measures] | 5642 | Advanced |
| 7 | exp *epilepsy/ or exp *obesity/ or exp *liver diseases/ or exp *kidney diseases/ or exp *heart diseases/ or exp *hiv infections/ or exp *neoplasms/ or exp *stroke/ | 81007 | Advanced |
| 8 | or/3-7 | 128657 | Advanced |
| 9 | 8 and coach*.mp. [mp=title, abstract, heading word, table of contents, key concepts, original title, tests & measures] | 268 | Advanced |
| 10 | 2 or 9 | 427 | Advanced |
| 11 | (epilep* or obese or obesity or liver or hepat* or kidney or renal or nephr* or heart or cardiac or coronary or myocard* or hiv or cancer or stroke*).mp. [mp=title, abstract, heading word, table of contents, key concepts, original title, tests & measures] | 219172 | Advanced |
| 12 | 11 and coach*.mp. [mp=title, abstract, heading word, table of contents, key concepts, original title, tests & measures] | 422 | Advanced |
| 13 | 12 and (health promotion/ or behavior*.mp. or behaviour*.mp. or change*.mp. or lifestyle.mp. or "life style".mp.) [mp=title, abstract, heading word, table of contents, key concepts, original title, tests & measures] | 244 | Advanced |
| 14 | ((life or lifestyle or health or behavior* or wellness or peer or recover*) adj3 coach*).mp. [mp=title, abstract, heading word, table of contents, key concepts, original title, tests & measures] | 1435 | Advanced |
| 15 | (8 or 11 or chronic*.mp.) and 14 [mp=title, abstract, heading word, table of contents, key concepts, original title, tests & measures] | 176 | Advanced |
| 16 | 10 or 13 or 15 | 574 | Advanced |
| 17 | limit 16 to yr="2005 - 2016" | 499 | Advanced |
| 18 | 17 and (study or studies or cohort* or intervention* or prospective* or retrospective* or "cross section*").mp. [mp=title, abstract, heading word, table of contents, key concepts, original title, tests & measures] | 405 | Advanced |
| 19 | 17 and random*.mp. [mp=title, abstract, heading word, table of contents, key concepts, original title, tests & measures] | 145 | Advanced |
| 20 | limit 17 to (clinical study or clinical trial, all or clinical trial, phase i or clinical trial, phase ii or clinical trial, phase iii or clinical trial, phase iv or clinical trial or comparative study or controlled clinical trial or evaluation studies or meta analysis or multicenter study or observational study or pragmatic clinical trial or randomized controlled trial or systematic reviews or validation studies) [Limit not valid in PsycINFO; records were retained] | 3 | Advanced |
| 21 | 18 or 19 or 20 | 412 | Advanced |
| 22 | limit 21 to all journals | 338 |  |

CINAHL

| **#** | **Query** | **Limiters/Expanders** | **Last Run Via** | **Results** |
| --- | --- | --- | --- | --- |
| S16 | S13 AND S14 | Limiters - Published Date: 20050101-20151231; Exclude MEDLINE records; Age Groups: All Adult  Search modes - Boolean/Phrase | Interface - EBSCOhost Research Databases  Search Screen - Advanced Search  Database - CINAHL with Full Text | 18 |
| S15 | S13 AND S14 | Search modes - Boolean/Phrase | Interface - EBSCOhost Research Databases  Search Screen - Advanced Search  Database - CINAHL with Full Text | 458 |
| S14 | "coach*" | Search modes - Boolean/Phrase | Interface - EBSCOhost Research Databases  Search Screen - Advanced Search  Database - CINAHL with Full Text | 4,659 |
| S13 | S1 OR S2 OR S3 OR S4 OR S5 OR S6 OR S7 OR S8 OR S9 OR S10 OR S11 OR S12 | Search modes - Boolean/Phrase | Interface - EBSCOhost Research Databases  Search Screen - Advanced Search  Database - CINAHL with Full Text | 640,227 |
| S12 | (MH "Neoplasms+") | Search modes - Boolean/Phrase | Interface - EBSCOhost Research Databases  Search Screen - Advanced Search  Database - CINAHL with Full Text | 200,896 |
| S11 | (MH "Asthma+") | Search modes - Boolean/Phrase | Interface - EBSCOhost Research Databases  Search Screen - Advanced Search  Database - CINAHL with Full Text | 20,237 |
| S10 | (MH "Stroke+") | Search modes - Boolean/Phrase | Interface - EBSCOhost Research Databases  Search Screen - Advanced Search  Database - CINAHL with Full Text | 35,916 |
| S9 | (MH "Cardiovascular Diseases+") | Search modes - Boolean/Phrase | Interface - EBSCOhost Research Databases  Search Screen - Advanced Search  Database - CINAHL with Full Text | 265,364 |
| S8 | (MH "Liver Diseases+") | Search modes - Boolean/Phrase | Interface - EBSCOhost Research Databases  Search Screen - Advanced Search  Database - CINAHL with Full Text | 23,258 |
| S7 | (MH "Obesity+") | Search modes - Boolean/Phrase | Interface - EBSCOhost Research Databases  Search Screen - Advanced Search  Database - CINAHL with Full Text | 40,563 |
| S6 | (MH "Epilepsy+") | Search modes - Boolean/Phrase | Interface - EBSCOhost Research Databases  Search Screen - Advanced Search  Database - CINAHL with Full Text | 7,299 |
| S5 | (MH "Osteoarthritis+") | Search modes - Boolean/Phrase | Interface - EBSCOhost Research Databases  Search Screen - Advanced Search  Database - CINAHL with Full Text | 11,390 |
| S4 | (MH "Arthritis+") | Search modes - Boolean/Phrase | Interface - EBSCOhost Research Databases  Search Screen - Advanced Search  Database - CINAHL with Full Text | 31,645 |
| S3 | (MH "Hypertension+") | Search modes - Boolean/Phrase | Interface - EBSCOhost Research Databases  Search Screen - Advanced Search  Database - CINAHL with Full Text | 34,419 |
| S2 | (MH "Diabetes Mellitus+") | Search modes - Boolean/Phrase | Interface - EBSCOhost Research Databases  Search Screen - Advanced Search  Database - CINAHL with Full Text | 75,582 |
| S1 | (MH "Chronic Disease") OR (MH "Pulmonary Disease, Chronic Obstructive+") OR (MH "Renal Insufficiency, Chronic+") | Search modes - Boolean/Phrase | Interface - EBSCOhost Research Databases  Search Screen - Advanced Search  Database - CINAHL with Full Text |  |

SCOPUS

( ( TITLE-ABS-KEY ( "chronic disease*"  OR  hiv  OR  epilep*  OR  diabetes*  OR  hypertension*  OR  copd  OR  "chronic obstructive"  OR  asthma*  OR  arthrit*  OR  osteoarthrit*  OR  obesity  OR  obese  OR  liver  OR  hepat*  OR  renal  OR  kidney  OR  heart  OR  cardiac  OR  stroke  OR  myocardial  OR  cancer*  OR  carcinoma*  OR  neoplasm* )  AND  coach*  AND  adult*  AND  PUBYEAR  >  2004 )  AND  ( life  OR  lifestyle  OR  health  OR  behavior*  OR  behaviour*  OR  health  OR  wellness  OR  peer  OR  recover*  OR  chang*  OR  promot* )  AND NOT  ( PMID ( 1*  OR  2*  OR  3*  OR  4*  OR  5*  OR  6*  OR  7*  OR  8*  OR  9* ) ) )  AND  ( study  OR  studies  OR  correlat*  OR  associat*  OR  compar*  OR  systematic*  OR  cohort  OR  intervention*  o )  AND  ( LIMIT-TO ( DOCTYPE ,  "ar" )  OR  LIMIT-TO ( DOCTYPE ,  "re" ) ) 826

Ovid

Database(s): Embase 1988 to 2016 Week 06, Ovid MEDLINE(R) In-Process & Other Non-Indexed Citations and Ovid MEDLINE(R) 1946 to Present, PsycINFO 1987 to February Week 1 2016, EBM Reviews - Cochrane Central Register of Controlled Trials January 2016
Search Strategy:

| **#** | **Searches** | **Results** |
| --- | --- | --- |
| 1 | exp Health Promotion/ | 156180 |
| 2 | coach*.mp. and 1 | 862 |
| 3 | ((clinical or life or lifestyle or "life style" or health or behavior* or behaviour* or wellness or peer or recover* or change or activit*) adj3 coach*).mp. | 4693 |
| 4 | 2 or 3 | 5160 |
| 5 | exp Health Facilities/ | 1685995 |
| 6 | exp Primary Health Care/ | 243696 |
| 7 | (((health* or care or medical or rehabilitation) adj2 (facility or facilities or unit or units or center*)) or "ambulatory care" or "birthing center*" or clinic or clinical or clinics or hospital* or "primary care" or "primary health care" or "primary healthcare" or "primary medical care").mp. | 12670003 |
| 8 | 5 or 6 or 7 | 12944670 |
| 9 | 4 and 8 | 1875 |
| 10 | exp Randomized Controlled Trial/ | 782546 |
| 11 | exp triple blind procedure/ | 117 |
| 12 | exp Double-Blind Method/ | 365378 |
| 13 | exp Single-Blind Method/ | 57286 |
| 14 | exp latin square design/ | 321 |
| 15 | exp Placebos/ | 292733 |
| 16 | exp Placebo Effect/ | 8541 |
| 17 | ((randomized adj3 study) or (randomized adj3 trial) or (randomised adj3 study) or (randomised adj3 trial) or "pragmatic clinical trial" or (doubl* adj blind*) or (doubl* adj mask*) or (singl* adj blind*) or (singl* adj mask*) or (tripl* adj blind*) or (tripl* adj mask*) or (trebl* adj blind*) or (trebl* adj mask*) or "latin square" or placebo* or nocebo* or random*).mp,pt. | 3184394 |
| 18 | or/10-17 | 3184394 |
| 19 | 9 and 18 | 756 |
| 20 | from 9 keep 856-1357 | 502 |
| 21 | limit 20 to (randomized controlled trial or pragmatic clinical trial) [Limit not valid in Embase,PsycINFO,CCTR; records were retained] | 127 |
| 22 | 19 or 21 | 756 |
| 23 | limit 22 to ("all adult (19 plus years)" or "young adult (19 to 24 years)" or "adult (19 to 44 years)" or "young adult and adult (19-24 and 19-44)" or "middle age (45 to 64 years)" or "middle aged (45 plus years)" or "all aged (65 and over)" or "aged (80 and over)") [Limit not valid in Embase,PsycINFO,CCTR; records were retained] | 682 |
| 24 | limit 23 to (adult <18 to 64 years> or aged <65+ years>) [Limit not valid in Ovid MEDLINE(R),Ovid MEDLINE(R) In-Process,PsycINFO,CCTR; records were retained] | 510 |
| 25 | limit 22 to ("all infant (birth to 23 months)" or "all child (0 to 18 years)" or "newborn infant (birth to 1 month)" or "infant (1 to 23 months)" or "preschool child (2 to 5 years)" or "child (6 to 12 years)" or "adolescent (13 to 18 years)") [Limit not valid in Embase,PsycINFO,CCTR; records were retained] | 602 |
| 26 | limit 25 to (embryo or infant or child or preschool child <1 to 6 years> or school child <7 to 12 years> or adolescent <13 to 17 years>) [Limit not valid in Ovid MEDLINE(R),Ovid MEDLINE(R) In-Process,PsycINFO,CCTR; records were retained] | 295 |
| 27 | 26 not 24 | 47 |
| 28 | 22 not 27 | 709 |
| 29 | from 28 keep 481-709 | 229 |
| 30 | (newborn* or neonat* or infant* or toddler* or child* or adolescent* or paediatric* or pediatric* or girl or girls or boy or boys or teen or teens or teenager* or preschooler* or "pre-schooler*" or preteen or preteens or "pre-teen" or "pre-teens" or youth or youths).mp. | 7083713 |
| 31 | (adult or adults or "middle age" or "middle aged" or elderly or geriatric*).mp. | 12155728 |
| 32 | 30 not 31 | 4196615 |
| 33 | 29 not 32 | 210 |
| 34 | from 28 keep 1-480 | 480 |
| 35 | 33 or 34 | 690 |
| 36 | limit 35 to yr="1990 -Current" | 689 |
| 37 | limit 36 to (editorial or erratum or letter or note or addresses or autobiography or bibliography or biography or blogs or comment or dictionary or directory or interactive tutorial or interview or lectures or legal cases or legislation or news or newspaper article or overall or patient education handout or periodical index or portraits or published erratum or video-audio media or webcasts) [Limit not valid in Embase,Ovid MEDLINE(R),Ovid MEDLINE(R) In-Process,PsycINFO,CCTR; records were retained] | 1 |
| 38 | 36 not 37 | 688 |
| 39 | remove duplicates from 38 | 395 |

Scopus

1. TITLE-ABS-KEY(((clinical or life or lifestyle or "life style" or health or behavior* or behaviour* or wellness or peer or recover* or change or activit*) W/3 coach*))
2. TITLE-ABS-KEY(((health* or care or medical or rehabilitation) W/2 (facility or facilities or unit or units or center*)) OR "ambulatory care" OR "birthing center*" OR clinic OR clinical OR clinics OR hospital* OR "primary care" OR "primary health care" OR "primary healthcare" OR "primary medical care")
3. TITLE-ABS-KEY((randomized W/3 study) OR (randomized W/3 trial) OR (randomised W/3 study) OR (randomised W/3 trial) OR "pragmatic clinical trial" OR (doubl* W/1 blind*) OR (doubl* W/1 mask*) OR (singl* W/1 blind*) OR (singl* W/1 mask*) OR (tripl* W/1 blind*) OR (tripl* W/1 mask*) OR (trebl* W/1 blind*) OR (trebl* W/1 mask*) OR "latin square" OR placebo* OR nocebo* OR random*)
4. PUBYEAR AFT 1989
5. 1 and 2 and 3 and 4
6. TITLE-ABS-KEY(newborn* or neonat* or infant* or toddler* or child* or adolescent* or paediatric* or pediatric* or girl or girls or boy or boys or teen or teens or teenager* or preschooler* or "pre-schooler*" or preteen or preteens or "pre-teen" or "pre-teens" or youth or youths) AND NOT TITLE-ABS-KEY(adult or adults or "middle age" or "middle aged" or elderly or geriatric*)
7. 5 and not 6
8. DOCTYPE(le) OR DOCTYPE(ed) OR DOCTYPE(bk) OR DOCTYPE(er) OR DOCTYPE(no) OR DOCTYPE(sh)
9. 7 and not 8
10. PMID(0*) OR PMID(1*) OR PMID(2*) OR PMID(3*) OR PMID(4*) OR PMID(5*) OR PMID(6*) OR PMID(7*) OR PMID(8*) OR PMID(9*)
11. 9 and not 10
